# Consensus workshops on the development of an ADHD medication management protocol using QbTest: developing a clinical trial protocol with multidisciplinary stakeholders

**DOI:** 10.1186/s12874-019-0772-2

**Published:** 2019-06-18

**Authors:** Charlotte L. Hall, Susan Brown, Marilyn James, Jennifer L. Martin, Nikki Brown, Kim Selby, Julie Clarke, Laura Williams, Kapil Sayal, Chris Hollis, Madeleine J. Groom

**Affiliations:** 1 Division of Psychiatry and Applied Psychology, Institute of Mental Health, University of Nottingham Innovation Park, Triumph Road, Nottingham, NG7 2TU UK; 2 Division of Rehabilitation and Ageing, University of Nottingham, Nottingham, UK; 3 Department of Community Paediatrics, Medway NHS Foundation Trust, Kent, UK; 4 Acting Consultant Community Paediatrics, Grantham and District Hospital, United Lincolnshire Hospitals NHS Trust, Grantham, UK; 5 Child and Adolescent, Developmental Psychiatry, School of Medicine, University of Nottingham and CANDAL (Centre for ADHD and Neuro-developmental Disorders across the Lifespan), Institute of Mental Health, University of Nottingham Innovation Park, Triumph Road, Nottingham, NG7 2TU UK; 6 Devlopmental Psychiatry Queens Medical Centre, School of Medicine, and MindTech, Institute of Mental, University of Nottingham, Innovation Park, Triumph Road, Nottingham, NG7 2TU UK

**Keywords:** Protocol development, Expert workshop, QbTest, Medication, Management, Titration, Patient and public involvement

## Abstract

**Background:**

The study design and protocol that underpin a randomised controlled trial (RCT) are critical for the ultimate success of the trial. Although RCTs are considered the gold standard for research, there are multiple threats to their validity such as participant recruitment and retention, identifying a meaningful change, and non-adherence to the protocol. For clinical RCTs, involving patients and clinicians in protocol design provides the opportunity to develop research protocols that are meaningful to their target audience and may help overcome some of the inherent threats in conducting RCTs. However, the majority of protocols do not describe the methodology underpinning their development, limiting the amount of learned experience shared between research groups.

**Method:**

With the purpose of reporting a collaborative approach towards developing a protocol, we present the findings from three sequential workshops that were conducted with the aim of developing a protocol to investigate the feasibility of adding a computerised test of attention, impulsivity and activity (QbTest) to medication management of children and young people with Attention deficit hyperactivity disorder (ADHD). Based on previous qualitative interviews with clinicians and families, each workshop prioritised topics for focused discussion. Information from the workshops was fed back to the participants for reflection in advance of the next workshop.

**Results:**

The workshops involved 21 multi-disciplinary ADHD experts, including clinicians, patient and public involvement (PPI) members, parents of young people with ADHD and researchers. The consensus workshops addressed key research issues such as: the most relevant outcome measures/ resource drivers; methods and time points for data collection; and the clinical protocol for utilising the QbTest, including when best to use this within the medication management process. The resulting protocol details a feasibility RCT design describing these factors.

**Conclusion:**

Protocols which are co-developed may help overcome some of the risks associated with RCT completion (e.g. recruitment, retention, protocol adherence) and help prioritise outcomes of greater relevance to the populations under study. The methodology has potential value for researchers and organisations developing clinical guidelines, and offers insights into the valuable impact of PPI upon trial design.

**Trial registration:**

Clinicaltrials.gov NCT03368573, 11th December 2017 (retrospectively registered).

## Background

Although every clinical trial should be underpinned by a rigorously developed protocol [1] there is little published evidence or guidance on the development of randomised controlled trial (RCT) protocols. Tezlaff et al. [1] outline the range of stakeholders for whom well-constructed protocols are pivotal, which include: trial participants, researchers, ethics committees, research funders and systematic reviewers. With the exponential growth of evidence-based medicine since the term was first coined in the early 1990s [2], there has been increasing acknowledgment and preference for RCTs [3], which are often advocated as the ‘gold standard’ in research designs. However, there are multiple, well-reported pitfalls in conducting RCTs, including: low participant recruitment, differential drop-out across study arms, participants not wishing to be randomised, developing a suitable control intervention, identifying a meaningful change (that is of importance to individuals seeking treatment), and non-adherence to the protocol [4, 5]. In light of this, there is growing recognition of the need to involve patients and clinicians to improve the feasibility and acceptability of trial protocols, develop protocols which are meaningful to their target audience, and reduce the risk of trial failure. Indeed, the importance of patient and public involvement (PPI) in the early stages of trial design is recognised by major funding bodies across Europe and America, and is highlighted in National Institute for Health and Care Excellence (NICE) [6] guidelines.

PPI is increasingly recognised as a crucial part of research, and is now a condition of funding for most (if not all) health research within the UK. Inclusion of PPI goes beyond simply improving trial design and conduct (although this is one of its benefits), rather it reflects the rights of citizens to have a say in research that might affect their lives [7]. When meaningful PPI is achieved, it can have a positive impact on the design, conduct and success of research (including RCTs) [8]. Inclusion of PPI in the research process can be challenging for various reasons including experience and training of researchers, differing expectations (amongst researchers and PPI members) about the processes underpinning PPI, and its scope and purpose [9]. Methods of involvement (and its subsequent impact) vary widely, and there is increasing emphasis placed on the need to both adequately report PPI (with new guidance emerging to do this) [10], and assess its impact. Evidence is slowly building about its impact – both positive and negative – on the research process, research outcomes, and the experience of both researchers and PPI members themselves (see PiiAF guidance [11]). In the spirit of elucidating the impact of PPI as one of a range of voices in the protocol design process, this paper describes the development of a trial protocol with attention paid to the needs, opinions and rationale of the different expert groups that fed into it.

Over the past few years there has been an increased recognition of the importance of standardised reporting of clinical trial protocols. To this end, the SPIRIT (Standard Protocol Items: Recommendations for Interventional Trials) guidelines [12] were developed through a series of Delphi consensus surveys, consensus workshops and a systematic review of the literature. The primary aim of the SPIRIT checklist was to increase the rigour of reporting of trial protocols, however, the majority of protocols do not describe their methodological development [1]. Providing details of how the protocol was shaped and developed facilitates the opportunity for critical appraisal of the trial design and provides a platform for the sharing of learned-experiences across research groups. This is critical for the improvement of trial designs. It also highlights the potential impact of PPI on trial design, which is increasingly important to demonstrate.

The aim of this paper is to report the methodological development of the protocol for a feasibility study, the ‘QbTest Utility in Optimising Treatment for ADHD’ (QUOTA) study [13]. The study aims to develop a medication management for stimulant medication and research protocol for evaluation of the QbTest (described in more detail below), and investigate the feasibility and acceptability of the protocol when delivered in routine healthcare services for children with attention deficit hyperactivity disorder (ADHD) in the UK. To improve outcomes, NICE ADHD guidelines recommend regular monitoring of symptoms when children commence medication [14]. However, research suggests that monitoring can be difficult in routine practice [15] and clinicians often rely on subjective information such as reports from parents and teachers to ascertain improvement [16]. These sources can be unreliable and difficult to obtain. The addition of an objective test of attention and activity (QbTest) may improve the objectivity, reliability and speed of clinical decision-making [17–21], and so reduce the time to identify the optimal medication dose. The QbTest combines a computer test of attention and impulsivity with a measure of activity in order to assess the three core symptom domains of ADHD: attention, impulsivity and activity. The QbTest takes approximately 20-min to complete and provides an instant report which graphically displays summary score relevant to each symptom domain (inattention, hyperactivity, impulsivity) with reference to a large age- and gender-stratified normative database. This report is available to download from the Qbtech server within minutes of completion of the test. The clinician is then able to discuss the test report immediately with the family, or book a separate appointment to discuss the report alongside the results of other questionnaire measures. Further details on the QbTest can be found elsewhere [20]. Although research has indicated the utility of the test to aid assessment and management of ADHD [17–19, 22–24], there is a need to develop a standardised protocol for its use in routine clinical settings, in line with Medical Research Council (MRC) guidance [25], as well as to evaluate the feasibility of the protocol before proceeding to a fully-powered clinical trial. To enable this, the protocol for QUOTA was developed through workshops comprised of stakeholders and experts in ADHD. The aim of this paper is not to evaluate the protocol per se*,* but to describe the approach to co-creating a research protocol with multi-disciplinary members. By reporting and reflecting upon the key stages involved in these workshops, the resulting decisions and the rationale under-pinning them, and the perspectives that they reflect, this paper aims to inform the development of future research protocols for RCTs of clinical interventions.

## Method

The study team initially considered seeking expert opinion through a Delphi method. However, due to time and budgetary constraints, a workshop approach with multiple expert stakeholders was considered the most suitable option. Additionally, the team felt a workshop approach would allow greater interaction and discussion/reflection between members.

### Workshop members

Members of the research team, who were study co-applicants (MG, CLH, NB) invited 21 experts to attend three workshops. Members were selected to ensure representation from key stakeholders. The experts included four PPI members (parents of young people with ADHD whose children had received medication; including PPI co-applicant and co-author NB), one education expert with experience of using QbTest in schools, two representatives and clinical advisors from Qbtech (on hand to answer technical questions only), one health economics expert with prior experience of ADHD studies, nine healthcare professionals from a range of backgrounds (including five consultant child and adolescent psychiatrists, three consultant paediatricians and one nurse specialist - including co-authors CH, KS, KSe, JC), three academic team members (MG, CLH, MJ), and two representatives from National Institute for Health Research (NIHR) MindTech MedTech Co-operative (MindTech) (JLM and SB), who acted as facilitators and note-takers and also brought additional PPI expertise to the workshop design. Not all experts attended every workshop. The experts were informed that the aim of the workshops was to develop a medication protocol around QbTest, including selecting key outcomes and resource drivers that affect the child and their family. The feasibility of the protocol would then be evaluated in a feasibility RCT design, with the comparator being assessment-as-usual. The finalised protocol for the QUOTA study received ethical approval from West of Scotland Research Ethics Committee (REC) 1 REC reference 17/WS/0209.

### Selection of initial topics for discussion

The topics discussed at the workshops were based on thematic analysis of interview data collected from clinicians and parents who had previously taken part in an RCT of QbTest (this method and findings have been reported elsewhere [12]). These interviews revealed a clinical need to develop a more standardised approach to medication management using QbTest. In particular, they highlighted areas of uncertainty around; a) how, why and when QbTest should be used in medication management, b) how the optimum dose should be defined, and c) which outcomes are most meaningful to clinicians and families. One workshop was therefore dedicated to explore each of these topics resulting in a total of three workshops. When designing the workshops, content and format was driven by researchers, in collaboration with a PPI co-applicant (NB) who had previous experience of PPI in ADHD research studies.

At the time the workshops were designed, the application had undergone approval by the funding body (NIHR) to ensure it was of acceptable scientific rigour. It should be noted that not all study parameters were open for group discussion. For example, the choice of QbTest was not open for discussion, although other similar tests are available, for a review see Hall et al. [18]. The researchers also ensured that the evolving design was of sufficient quality and was within the boundaries of a feasibility RCT. Furthermore, clinical decisions around dose and titration must always sit within current best practice (i.e. NICE [6] guidelines) and follow the specified dosing schedule recommended for the drug. With this in mind, although the protocol was limited to stimulant medication, the choice of stimulant and its dosing schedule was left to clinicians. The focus was specifically on the use of QbTest in everyday practice, which may vary from clinician-to-clinician. In sum, the workshops were designed to address specific details of the protocol whilst recognising that some elements of the research design and some aspects of clinical practice, cannot be changed.

### Format of workshops

All workshops were held at the Institute of Mental Health at the University of Nottingham in England. The workshops took place between April – June 2017, with one workshop being held every month. Each workshop was scheduled to last for two hours.

Workshops were chaired by the Chief Investigator (MG) and facilitated by a researcher outside the study team (JLM). The facilitator’s primary role was to ensure the voices of each member were heard, paying particular attention to the inclusion of PPI members. The discussions of the meetings were noted by research team members CLH and SB as well as by JLM. The notes were collated and summarised by CLH and checked for accuracy by SB before being fed back to the group by email for consideration and reflection in advance of the next workshop.

In the first two workshops, members were initially divided into two groups to aid discussions and ensure each member had the chance to express their opinion. The groups were divided to ensure mixed composition of expertise. Both groups fed back their discussions to the entire workshop after initial discussions were completed, allowing for further discussion, particularly in relation to any areas of disagreement or uncertainty. In the second workshop, the choice was made to have the groups discuss different topics due to time constraints, as there was a need to cover sufficient content within the two hour workshop. In order to ensure wide input and ‘consensus’, each group fed back their discussion to the other, including describing the rationale behind decisions, and this was followed by facilitated discussion to debate and refine decision making.

In the third (and final) workshop, members remained as one group due to the comparatively low attendance rate and the need to reflect as a whole on the final protocol. The research team collated decisions and main discussion points after each workshop and fed this back to all members (regardless of whether they attended) between workshops. The start of the next workshop began with a reflection of the discussions from the previous workshop and the opportunity for members to raise any further discussion points.

## Results

Attendance at each workshop varied from 13 to 17 members (62–81-% of those invited), but always included at least four PPI members, a clinician and representatives from the research team and MindTech. Participants who could not attend were updated on the group discussion via telephone or email by the Trial Manager.

### Workshop 1: introduction and defining the medication protocol

The aim of the first workshop was to reach consensus on the medication protocol and therefore how best to use QbTest alongside current clinical practice to monitor the effects of stimulant medication.

The expert group (*n* = 15) consisted of six clinical members, four PPI members, two researchers, two MindTech representatives (as facilitator and note-taker) and one clinical advisor from Qbtech. The group was divided into two small groups consisting of seven members per group, which included at least one representative from each role (e.g. PPI, clinician, researcher). The additional member was the clinical advisor to Qbtech, who did not participate in group decision-making (to avoid conflicts of interest), and was present to provide expert advice on the use of the QbTest if required. The groups were informed of the aim of the workshop and given two points of guidance in developing the protocol: 1) the medication protocol must include a baseline assessment of symptoms prior to medication initiation; 2) follow-up assessments should be within 12-weeks from baseline (due to restrictions imposed by funding limitations). No other guidance was given. The group were encouraged to develop a feasible and acceptable protocol based on their expertise in ADHD. In order to provide some anchor points for discussion, the groups were set three main questions to answer.

Q1: When should the first follow-up QbTest (on medication) take place?

The groups discussed when the first (baseline) QbTest should be conducted. One clinician noted there is no ‘gold standard’ in assessing ADHD, and as such there would likely be variation in clinical practice, with some children already receiving a QbTest as part of their diagnostic assessment. Both clinicians and PPI members raised concerns that if the child had already received a QbTest during their initial diagnostic assessment it would not be appropriate to attend an additional consultation to repeat the test purely for the purpose of a baseline assessment before starting medication. Conversely, clinicians also raised concerns that if the diagnostic assessment had taken place several weeks or months previously, the QbTest may be ‘out of date’ and not reflect current symptomatology. The Qbtech clinical advisor was brought into the group to provide advice on the reliability of QbTest results over repeated testing. It was also noted by PPI members that there should, where possible, be no delay in medication initiation as a result of participating in a trial, and minimisation of additional appointments due to difficulties gaining permission for time away from school. Thus, any clinic baseline measure (and consent) should be collected at the point the family consent to take part in the study.

With regard to when the first follow-up QbTest should be conducted, group one considered that 1–2 weeks after medication initiation was ideal. In this group, the clinical experts noted the importance of identifying early non- responders or partial responders to medication in order to switch medications or increase dosages to facilitate early optimal responding. This was supported by PPI members who noted that they had often experienced a substantial delay in receiving the ADHD diagnosis and were keen to receive the most appropriate medication as soon as possible. In contrast, the second group had selected a longer first follow-up of 6–8 weeks after medication initiation. Although this group noted the importance of a telephone review two weeks after starting medication, the clinicians in this group raised concerns with the feasibility for clinics of conducting a face-to-face follow-up assessment after two weeks. PPI members in this group echoed the sentiments of those in group one, but they also noted that they felt their child may be reluctant to sit two QbTests in quick succession.

When the two groups presented their discussions back to the whole workshop there were a range of critical factors discussed, including the need to monitor side-effects quickly (an issue raised by both PPI members and clinicians), to be able to adjust dose or switch to an alternative medication as soon as possible if there was a sub-optimal response (important to both PPI and clinicians), and the issue of limited clinic appointments available and/or families being unwilling to attend clinic too frequently. All members were in unanimous agreement that there would need to be some early checking of adverse effects, and it was discussed this could be done by the child’s General Practitioner (GP). However, PPI members raised concern about getting access to GP appointments and research team members noted that it would be difficult to make this a standard part of the study protocol without involving GPs directly in the research. Critically, after group reflection, it was agreed by all members that having the first follow-up QbTest conducted at 6–8 weeks would not allow for early identification of sub-optimal responses, and could lead to a lengthy titration schedule.

#### Recommendations

- If children have received a QbTest as part of diagnostic assessment within 12 weeks before medication initiation, no additional baseline QbTest will be conducted. This diagnostic QbTest will be used as the baseline assessment against which to monitor medication effects.
- The first follow-up QbTest would be conducted 2–4 week after baseline. This time frame was selected through group discussion to allow early monitoring of adverse effects and sub-optimal response, but provide a sufficient time window for organising clinic appointments.

Q2: After the first follow-up assessment how frequently should QbTest be repeated?

The need for QbTest to provide insight into the efficacy of medication was counterbalanced by the additional demand this places on clinicians and families. There was consensus from all members that performing a QbTest assessment at each dose titration would not be feasible. Group one felt that two follow-up QbTests could be conducted within the 12-week period (one at 1–2 weeks after medication initiation and one at approximately 8–10 weeks), whereas during their initial discussions group two had felt that only one follow-up QbTest was required (at 6–8 weeks with a telephone review at 2 weeks). The combined group agreed that the clinician should not alter their medication review process apart from adding the QbTest, and thus supplementary telephone/clinic reviews could be conducted in accordance to the clinician’s standard practice. It was also noted that the control group may not be seen at all during the follow-up period and there was a need to balance any effect of additional clinic contact. The clinicians felt that arranging additional appointments to treatment as usual in the control group would be a clinical burden and not feasible, however, telephone contacts were viable. The final recommendations to resolve the conflicting opinions from group one and two were made through whole group discussion, and the decision was partly based on pragmatism and compromise between ‘best practice’ and ‘real world demands’. After this whole group discussion, both groups endorsed the recommendations.

#### Recommendations

- After whole group reflection, it was agreed that a second follow-up QbTest on medication should be conducted within an 8–10 week period (and no more than 12 weeks), to provide an additional opportunity to check medication response. This would therefore lead to a protocol with one baseline assessment, one follow-up assessment 2–4 weeks after baseline/prescription of medication and a final follow-up 8–10 weeks after baseline/prescription of medication.
- There should be at least two contacts for participants in the control group (which could be conducted over the telephone during the follow-up period) to control for the additional assessments being conducted in the QbTest group.

Q3: How should we define the optimum dose?

Both group one and two expressed strong opinions that the optimum dose should not be defined by reaching a set score on a symptom rating scale. This was unanimous across PPI members and clinicians who felt that reaching a pre-defined score did not capture the complex nature of ADHD, nor the impact of the symptoms on school, family and the young person. Importantly, PPI members noted the desire to have a child who was a ‘true reflection of themselves’ and not over-medicated to the point they lost ‘their personality’ and ‘the things that make them who they are’; rather, an optimum dose would be one that reduced a child’s symptoms until the condition was ‘manageable’.

#### Recommendations

- The definition of ‘optimal dose’ from which no further adjustments are needed would be defined by the clinician integrating the opinions of the young person, the family and schools, alongside the QbTest report in the QbTest group.

### Workshop 2: study outcomes and instruments

The aim of the second workshop was to gain expert opinion on which outcome measures should be used in the research protocol and to ascertain the resource implications of introducing QbTest for the NHS and a broader societal perspective including the education sector and parents \ carers. It should be noted that for a feasibility RCT the focus is on the completion rates of outcome measures and the acceptability to the participants, rather than to collect sufficient data to conduct a fully-powered definitive answer on effectiveness.

Seventeen experts, including six clinicians, four PPI members, two researchers, two representatives from MindTech, two advisors from Qbtech, and one health economist, were divided into two groups. Group 1 (*n* = 8) primarily discussed possible outcome measures to assess change in symptom or global functioning. Group 2 (*n* = 9) primarily discussed issues surrounding implementing a health economic measure. Each group focussed on their assigned topic before feeding back to the group as a whole.

#### Selecting suitable measures of symptom outcome and global functioning

Group 1 were presented with a range of outcome measures typically used in ADHD research trials to guide their discussion. They were informed that the SNAP-IV (Swanson, Nolan, and Pelham Rating Scale [26]), a well-used measure of ADHD symptoms, had previously been selected by academic research team members as a highly valuable assessment to include in this study as part of the funding application. Therefore, the choice of this measure was not part of the discussion. The group were asked to discuss the possible use of six additional measures.

*Strengths and Difficulties Questionnaire (SDQ)* [27]. The SDQ provides a brief measure of behavioural and emotional difficulties and was considered important to use at baseline to help describe the core functioning and behaviours of the sample. However, clinical and PPI members unanimously agreed that the SNAP-IV comprehensively measured symptom change that was more relevant to ADHD.

*Children’s Global Assessment (CGAS)* [28]. The CGAS is a clinician completed measure of global functioning. The CGAS was seen by clinicians as being too generic, thus making it difficult to accurately complete. Both clinicians and PPI members felt the data would not be clinically meaningful due to this.

*Clinical Global Impressions (CGI)* [29]. The CGI is a clinician-completed assessment of symptom severity and improvement. Assessing these issues was seen as important by all members and the CGI was considered a quick and easy method to achieving this. Both clinical and PPI members felt that the clinician should complete this measure as part of their consultation with the family, reflecting joint rather than clinician-only judgements. However, all parties observed that the wording of the CGI was not always appropriate for children with ADHD and that clinicians should be careful to re-phrase the questions when discussing the CGI with the family. A clinician with research expertise also pointed out that completing the CGI throughout the intervention may influence clinicians’ practice, as such, it should only be completed at baseline and at the end of the intervention.

*EQ-5D-Y* [30]. The EQ-5D-Y is a widely used assessment of quality of life, comprised of a Likert scale and a Visual Analogue Scale (VAS). The health economist and research team members stressed the importance of having a measure of quality of life. All group members considered the VAS as a user-friendly way to capture how the young person felt in general. However, not all items (e.g. mobility) were believed to be relevant to ADHD, this was voiced by both clinicians and PPI members.

*Child Health Utility 9D* [31]. *The CHU9D is* a paediatric quality of life measure for use in health care resource-allocation decision making. This was discussed as an alternative to the EQ-5D-Y. The group unanimously felt the questions such as being ‘worried’ or ‘sad’ were more relevant and the questionnaire was deemed fit for inclusion.

*Adverse Effects Scale* [32]. This scale proved popular with all group members, covering the key issues surrounding the impact of medication. A clinical academic lead highlighted the importance of only the researchers having access to parent and young person completed versions of this scale so as not to influence practice. Alongside this, researchers noted the importance of having a brief measure of medication adherence, to ensure participants were taking their medication. Members felt this should be a simple question which asks over the past 4 weeks how often has the child taken their medication (every day, most days, occasionally, never/withdrawn).

*QbTest.* All group members stressed the importance of discussing the results of the QbTest with the family. Their previous experience revealed this was not always standard practice [19], and the need to interpret and discuss QbTest scores would be a key part of the study protocol. It was also discussed whether the control group should complete a QbTest at the second follow-up (at 8–10 weeks), with the result being withheld from the clinician until the participant exits the trial, so that the scores can be used as an outcome measure. However, PPI members felt it was not ethical to bring families in to sit the test without receiving the benefits of the results. Ultimately the PPI members’ concerns (around the burden of gathering extra data without it benefitting the child’s treatment) took precedence over the desire for additional data from the control group.

#### Recommendations

- SDQ should only be used to describe the sample characteristics at baseline rather than a measure of outcome.
- CGAS should not be included as a measure.
- CGI should be completed at baseline and at the end of the intervention through joint parent and clinical discussions.
- CHU9D rather than the EQ5DY should be used as the proxy of quality of life measure.
- Side effects scale should be included with an additional medical adherence question.
- QbTest would not be conducted in the control arm to monitor medication effects.

#### Capturing health and education resource use

Group 2 was co-led by the research team’s Health Economist (MJ). The group discussed issues related to capturing use of health services and used the Client Service Receipt Inventory (CSRI) [33] as a starting point for discussion. As an inventory, the CSRI allows researchers to flexibly select the items best suited to the needs of the individual research project.

The first issue centred on how often a measure of resource use should be completed in the trial. PPI members discussed difficulties in recall, but also the burden of completing the measure too frequently. They felt that some items would remain the same (such as the school the child attended) and over-duplication of the same information would be tiresome. However, other information such as attending numerous clinic appointments and seeing various healthcare professionals was difficult to remember and they may not know the professional background of their clinician (e.g. whether they have seen a nurse or a psychiatrist). A member of the research team suggested that participants could keep a diary of appointments. However, consensus was not reached on whether such an approach would capture the important information or be feasible to implement in this trial. The trial was not considered large enough to test both options (standard health resource measure completion vs. the diary method), but that this could be an avenue for future research.

The second issue considered who should complete the resource use questionnaire. The research team presented the group with a modified CSRI, with instructions that the items should only be viewed as starting point. Group members believed that ideally parents should complete this assessment, but the current format was complex and didn’t lend itself to self-completion in its standard format. In addition, its purpose was not immediately obvious from the listed items, and the PPI members said it risked resembling an assessment for state-funded financial aid which could deter parents from completing it. They felt that parents should receive a clear explanation from the researcher before its completion. PPI members also noted that they did not necessarily consider the economic impact of ADHD as an important outcome, seeing improvement in the symptoms as their main goal. Instead they identified costs around the family/relationships (such as parental stress and depression, parental sickness\absence from work, relationship counselling and divorce) as important. Some of these are not captured in a typical standard CSRI form and although they were considered important, it was outside the scope of this research to include these elements. Further research could investigate the feasibility of collecting such outcomes. Although financial costs, such as damage to household objects was noted by PPI members (and recorded on the CSRI), it was considered difficult to attribute a cost to these damages. Using the full CSRI inventory was not considered to capture factors most important to families, and there was concern the families would not complete the inventory as a result. As such, consensus was reached that a purposively designed targeted resource use questionnaire should preferably be administered as an investigator-based structured interview, with attention paid to explaining why this assessment is carried out as part of the study.

The third issue discussed the questions which asked about school resource use (such as receiving in-class support). PPI members and the education expert noted that they may not always know the exact school resources allocated to their child. Both these stakeholders felt that schools were best placed to answer these questions, and the health resource use should be split into two forms, one for school completion and one for the family. The education expert noted the importance of using the correct wording to describe school placements and suggested that, where available, schools should also provide the child’s Provision Map to accurately identify resource use. On the basis of these discussions two draft health resource use pro formas were created, the ‘Resource Use – Services in Education’ (RUSE) for school completion and the ‘Resource Use – Services for Health (RUSH)’ for families to complete (available on request from the corresponding author).

#### Recommendations

- Repetition should be minimised; the CSRI inventory should be used to inform a targeted resource use pro forma (RUSH) only collecting data items that cannot be retrieved from elsewhere in the study. To minimise participant burden, items that are not expected to change over time should not be included.)
- The targeted resource use questionnaire should be administered as an investigator- based structured interview, which can be done by telephone (with clear explanation as to its purpose).
- The targeted health resource data collection tools should be split into two forms, one for school completion (RUSE) and one for the family (RUSH).

### Workshop 3: selecting a primary outcome and recording clinical decision making

**Fig. 1:**
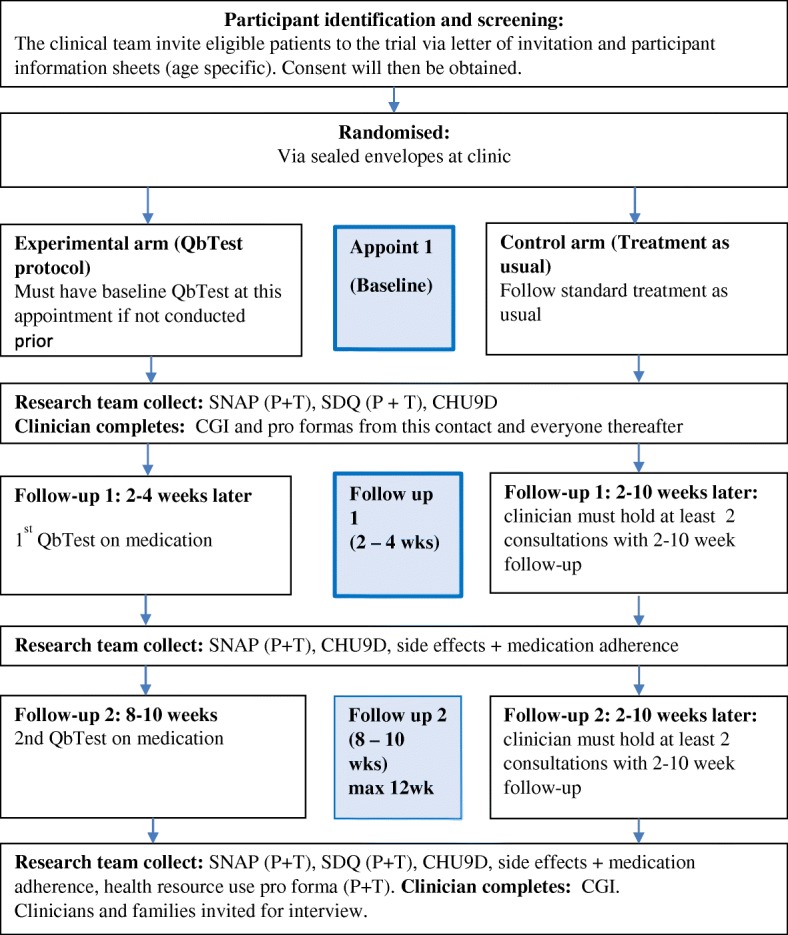
Study Flow

The last workshop had the lowest attendance (*n* = 13, 62%). Reasons for non-attendance included annual leave (4, 19%), prior clinical commitments (2, 9.5%), and feeling that the protocol was sufficiently developed (2, 9.5%). The aim of the final workshop was to reflect on the discussions, decide on a potential primary outcome and develop a clinician completed pro-forma. Thirteen members attended this workshop, including four clinicians, 4 PPI members, two researchers, two MindTech representatives and one advisor from Qbtech. The research team presented the proposed study flow to the team (Fig. 1).

PPI members and clinicians felt that general functioning at school had not been adequately addressed, therefore a decision was made to include both a parent- and teacher- completed SDQ at final follow-up to measure change in global mental health difficulties. Although this addition contrasts with the decision in workshop 1 *not* to have a second SDQ at final follow-up, upon further reflection the need to capture functioning at school was considered important and felt to be best captured by the SDQ, meriting inclusion as a secondary outcome measure. It was also noted that, where possible, the same teacher should complete the baseline and follow-up SDQs, but this was recognised to not always be possible if follow-ups span changes in school years.

#### Developing a clinician completed pro forma

The expert members were presented with a clinician pro forma used in a previous ADHD trial [20] which would be necessary to record clinical decision making throughout the trial. The members discussed issues around the need to capture: the frequency and length of appointments, the timing of QbTest, a formal record that the QbTest was discussed with the family, the methods used to diagnose the young person, other methods used to inform monitoring and medication decision-making, the drug and dose, and any other concomitant information. Clinicians noted the importance of having simple-to-complete questions using tick-boxes. A draft pro forma was created on the basis of this which underwent two rounds of further email discussions with the clinical members before a finalised pro forma was reached. The pro forma is included in Appendix 3. Additionally, clinicians felt that they were more likely to complete measures online than on paper as the latter would involve an additional step of posting results back to the research team. PPI members also felt online measure completion was easier, however, families should be offered the option of postal or telephone completion for all outcome measures to accommodate individual family preferences.

#### Selecting a primary outcome

The group were unanimous that a change on SNAP-IV score should be the primary outcome. Symptom improvement was considered of key importance for both clinicians and PPI members. The group discussed whether reaching a normalised score was the best option (as per workshop 1), however, as in the first workshop, some clinicians felt that was not achievable, and PPI members stressed the importance of having a child that was a true reflection of themselves, and being ‘normalised’ was not always desirable. Following the landmark Multimodal Treatment of ADHD (MTA) [34], a 25% reduction of symptoms was considered the most suitable primary outcome with normalization as secondary outcome for a future definitive RCT.

After discussion, both parents and clinicians felt that teachers were the best informants for completion of the SNAP-IV. Although the team discussed issues around potential low response rates from teachers, the clinicians and PPI members felt teachers were still the best informants (due to medication often being given for optimum effect during school hours and being able to compare with peers). The team concluded that teacher completion rates will be monitored as part of the QUOTA feasibility study to determine the suitability of teacher completed SNAP-IV as the primary outcome for the future RCT. The decisions made in the workshop, including the reasoning behind them, were circulated to non-attending members for their approval. All were in agreement.

#### Recommendations

- A parent and teacher completed SDQ should be included at final follow-up to gain a measure of general difficulties.
- A simple tick-box clinician completed pro forma should be recorded online.
- Change (of 25%) on SNAP-IV score should be the primary outcome.
- Teacher completion rates should be monitored as part of the QUOTA feasibility study to determine the suitability of teacher completed SNAP-IV in the full RCT.

## Discussion

The consensus workshops provided rich, multifaceted information to support the development of an ADHD medication management protocol for QbTest which would be tested in a feasibility RCT. Given the increasing awareness of the importance of involving patients, caregivers and public members in research trials [35], this methodologically-driven report has broad applicability to researchers working in all fields of clinically-facing research, but particularly for those working in ADHD and other related areas such as in the child mental health or neurodevelopment fields, who can draw upon the preferences expressed to inform future trial design.

It was interesting to note the broad consensus across both clinicians and PPI members in numerous areas, with agreement on the need to balance the burden of data collection against the need to gather important information. It is also noteworthy that many RCTs collect multiple secondary outcomes which may not be pertinent to the aim of the study. Our findings suggest these should be streamlined in future research. Furthermore, there was consensus across clinicians and PPI members that the importance of reaching a meaningful reduction in symptoms was more important than achieving a ‘normalised’ score. When selecting primary outcomes there can be a tendency for research teams to define clinically meaningful change from a clinician’s perspective, however, the opinion and lived experience of the patient and caregiver should inform this judgement.

Members of the consensus workshop were overwhelmingly positive about their experience of being part of the group, with each member feeling they had an opportunity to be heard. A protocol was developed on the basis of these workshops, which has been published [13]. The QUOTA study is in the early stages of participant recruitment so we cannot comment on whether recruitment was successful or protocol adherence was good as a result of this approach to its development. The QUOTA study will record and publish these outcomes as part of the feasibility assessment. It is recognised that it is difficult to record, measure and quantify the impact that involving patient and public members in research may have. To discern the impact that PPI members had in this instance, we have recorded where they contributed (and where they did not) which provides a route into charting their impact on decision making; logging of recommendations is a helpful way to capture impact [36]. It is not possible to know exactly how the protocol would have differed *without* PPI inclusion (as with the removal of any expert from the process) but mapping their specific contribution to decision making can help to reveal a footprint of sorts.

We now consider specific of the workshop design and identify possible areas for further research. One of the challenges of conducting the workshops was the need to balance opinions for ‘best practice’ with real-world time and finance constraints. There also remain challenges around whose viewpoint holds prominence when there are competing views. A key example is where the workshops highlighted outcomes that mattered to parents (relating to changes in the impact of the child’s ADHD on family life more generally) but would be difficult to capture and had less relevance to *clinical* outcomes. Given the constraints of the study, these were ultimately considered secondary to the other research aims (by the researchers, at least). However, this would be a useful development for future research in this field. Additionally, some points were raised (such as modifications to the methodology of completing economic measures through the diary method) which were outside the remit of this study to investigate. In terms of limitations, despite good representation across key stakeholders, our findings reflect the opinions of members who agreed to participate in the groups, who may be specifically motivated to develop a more closely-monitored titration process. Furthermore, the participants were invited through the study team’s known contacts in the field (including an ADHD support group for parents) and participation was not advertised. Additionally, it was not possible for children or young people to participate in the workshops. Although our invitation to participate was extended to both parents and their child, difficulties in organising groups outside of school hours and the nature of the workshops (several hours of focussed time), precluded young people from attending. Designs that provide a more child-friendly format would be useful in future to ensure the views of this group are included.

In the current study, each workshop comprised a slightly different format: two comprised small sub-groups (in one the same point was discussed and in the other the groups discussed separate points); the third involved whole-group discussion only. This was a reactive decision, rather than pre-planned, driven by the number of people available to attend each workshop (there were fewer people available for the third workshop) and time limitations. However, the choice was also strategic. The first workshop covered the most fundamental basic outline of the design on which consensus had to be obtained to allow progression; this was the most important element of its design and was given the most attention by making it the focus of discussion for both sub-groups. In the second workshop, although participants discussed different topics, they presented their discussions to the other group to ensure whole-group discussion. The third workshop (with no sub-groups) was designed to encourage discussion by all participants. At this stage, the members felt comfortable with all other members and thus smaller groups would not have been as necessary. Although maintaining the same format for every workshop would have led to greater consistency in the methodology, the more flexible approach chosen here ensured that the group format was best suited to the topics for discussion at each point and the group numbers at each stage. Further research could investigate the extent to which the structure and make-up of groups is best kept constant or should be made flexible to elicit information effectively.

It could be argued that a group approach can be less likely to elicit contradictory views or views that deviate from the norms being expressed, resulting in the most prevalent or forcefully expressed viewpoints dominating group discussion. To mitigate this, workshop facilitators were briefed in advance and instructed to give focus to PPI members as much as possible in order to seek their viewpoints in particular. Indeed, PPI co-applicant (NB) was involved in planning the workshops and was aware a key task would be to create a process that allowed PPI members to contribute freely. Furthermore, some of our PPI members were also partially chosen for their confidence and ability to express their opinion, and were able to create a welcoming environment to support other PPI members. Alternative methodological approaches include interviews with individual participants or a Delphi survey. Given the need to develop a study protocol that reflected the needs and views of different stakeholders, we felt the group method was preferable to individual interviews [36]. The Delphi method requires individual participants to answer questions/rate items and give feedback (usually remotely) which is collated by the research team. The protocol is then refined and participants are asked to re-rate each item. Although this approach ensures that individuals are not unduly influenced by a group-norm, it is timely and costly. Moreover, we felt that the information gathered through group discussions would be richer and enable a more meaningful consensus to be reached, driven by the stakeholders, rather than the researchers. Furthermore, built into the study design (but not part of the workshops) is a phase of qualitative data collection (workshops and interviews) exploring the experience of the protocol from clinician and family perspectives – both in a combined group setting and also individually in interviews – to draw out what its impact was in practice, and whether further adaptations to the protocol are required. So whilst the protocol design phase used group discussion alone, this sits within a broader inquiry that uses additional methods.

It should be noted that although this design was specific to designing a medication protocol for children, the concept of the formulation and delivery of the workshop, with relevant PPI members is pertinent for any trial involving human participants. We would also like to emphasise that although involving different stakeholders is an important and under-practiced part of research design, this should not be the exclusive process in designing a trial. Considerations for quality standards and scientific rigour must always be of paramount importance.

## Conclusions

By presenting the approach and findings of the workshops involving multidisciplinary members to develop a medication and trial protocol, we illuminate some of the consultative processes under-pinning the development of clinical protocols to enhance their acceptability and feasibility. By sharing our methodological approach, we hope to improve the transparency of protocol development and strongly advocate that researchers actively engage with clinical teams, industry, and patient and public members when developing protocols. Protocols which are co-developed may help overcome some of the barriers associated with successful completion of RCTs, such as issues with recruitment, retention and adherence. Our methodology and findings may also be useful for groups and organisations that develop clinical consensus guidelines, and for clinicians and researchers working in the field of ADHD in particular.

## Data Availability

Data sharing is not applicable to this article as no datasets were generated or analysed during the current study.

## Abbreviations

ADHD: Attention deficit hyperactivity disorder
CGAS: Children’s Global Assessment Scale
CGI: Clinical Global Impressions
CHU9D: Child Health Utility 9D
CSRI: Client Service Receipt Inventory
GP: General Practitioner
MRC: Medical Research Council
MTA: Multimodal Treatment of ADHD
NICE: National Institute for Health and Care Excellence
NIH: National Institute for Health
NIHR: National Institute for Health Research
PiiAF: Public involvement impact assessment framework guidance
PPI: Patient and Public Involvement
QUOTA: QbTest Utility in Optimising Treatment for ADHD
RCT: Randomised Controlled Trial
REC: Research Ethics Committee
RUSE: Resource Use – Services in Education’
RUSH: Resource Use – Services for Health
SDQ: Strengths and Difficulties Questionnaire
SNAP-IV: Swanson, Nolan and Pelham 4th version
SPIRIT: Standard Protocol Items: Recommendations for Interventional Trials

## References

[1] Tetzlaff JM, Moher D, Chan A-W (2012). Developing a guideline for clinical trial protocol content: Delphi consensus survey. Trials..

[2] Group E-BMW (1992). Evidence-based medicine. A new approach to teaching the practice of medicine. JAMA..

[3] Grossman J, Mackenzie FJ (2005). The randomized controlled trial: gold standard, or merely standard?. Perspect Biol Med.

[4] Fogg L, Gross D (2000). Focus on research methods-threats to validity in randomized clinical trials. Res Nurs Health.

[5] Neugebauer EA, Rath A, Antoine S-L, Eikermann M, Seidel D, Koenen C, Jacobs E, Pieper D, Laville M, Pitel S (2017). Specific barriers to the conduct of randomised clinical trials on medical devices. Trials..

[6] NICE. Patient and Public Involvement Policy**.** https://www.nice.org.uk/media/default/About/NICE-Communities/Public-involvement/Patient-and-public-involvement-policy/Patient-and-public-involvement-policy-November-2013.pdf. Accessed 23 Mar 2018.

[7] Involve. Briefing notes for researchers: involving the public in NHS, public health and social care research. https://www.involve.org.uk/resources. Accessed 12 Mar 2018.

[8] Brett J, Staniszewska S, Mockford C, Seers K, Herron-Marx S, Bayliss H: The PIRICOM Study: A systematic review of the conceptualisation, measurement, impact and outcomes of patients and public involvement in health and social care research. 2010. Warwick: University of Warwick, http://wrap.warwick.ac.uk/47235/.

[9] Wilson Patricia, Mathie Elspeth, Keenan Julia, McNeilly Elaine, Goodman Claire, Howe Amanda, Poland Fiona, Staniszewska Sophie, Kendall Sally, Munday Diane, Cowe Marion, Peckham Stephen (2015). ReseArch with Patient and Public invOlvement: a RealisT evaluation – the RAPPORT study. Health Services and Delivery Research.

[10] Staniszewska S, Brett J, Simera I, Seers K, Mockford C, Goodlad S, Altman D, Moher D, Barber R, Denegri S (2017). GRIPP2 reporting checklists: tools to improve reporting of patient and public involvement in research. Research Involvement and Engagement.

[11] Popay J, Collins M, Group wtPS: The public involvement impact assessment framework guidance. *Universities of Lancaster, Liverpool and Exeter;* 2014. http://piiaf.org.uk/documents/piiaf-guidance-jan14.pdf. Accessed 18 Mar 2018.

[12] Chan A-W, Tetzlaff JM, Gøtzsche PC, Altman DG, Mann H, Berlin JA, Dickersin K, Hróbjartsson A, Schulz KF, Parulekar WR (2013). SPIRIT 2013 explanation and elaboration: guidance for protocols of clinical trials. BMJ..

[13] Hall CL, James M, Brown S, Martin JL, Brown N, Selby K, Clarke J, Vijayan H, Guo B, Sayal K (2018). Protocol investigating the clinical utility of an objective measure of attention, impulsivity and activity (QbTest) for optimising medication management in children and young people with ADHD ‘QbTest utility for Optimising treatment in ADHD’(QUOTA): a feasibility randomised controlled trial. BMJ Open.

[14] NICE: Attention deficit hyperactivity disorder:diagnosis and managment of ADHD in children, young people and adults. *Clinical Guideline 72*. In*.* London: National Institute for Health and Clinical Excellence 2008.

[15] Kovshoff H, Williams S, Vrijens M, Danckaerts M, Thompson M, Yardley L, Hodgkins P, Sonuga-Barke EJ (2012). The decisions regarding ADHD management (DRAMa) study: uncertainties and complexities in assessment, diagnosis and treatment, from the clinician’s point of view. Eur Child Adolesc Psychiatry.

[16] Hall CL, Taylor JA, Newell K, Baldwin L, Sayal K, Hollis C (2016). The challenges of implementing ADHD clinical guidelines and research best evidence in routine clinical care settings: Delphi survey and mixed-methods study. Br J Psychiatry Open.

[17] Hall CL, Selby K, Guo B, Valentine AZ, Walker GM, Hollis C (2016). Innovations in practice: an objective measure of attention, impulsivity and activity reduces time to confirm attention deficit/hyperactivity disorder diagnosis in children–a completed audit cycle. Child Adolesc Mental Health.

[18] Hall CL, Valentine AZ, Groom MJ, Walker GM, Sayal K, Daley D, Hollis C (2016). The clinical utility of the continuous performance test and objective measures of activity for diagnosing and monitoring ADHD in children: a systematic review. Eur Child Adolesc Psychiatry..

[19] Hall CL, Valentine AZ, Walker GM, Ball HM, Cogger H, Daley D, Groom MJ, Sayal K, Hollis C (2017). Study of user experience of an objective test (QbTest) to aid ADHD assessment and medication management: a multi-methods approach. BMC Psychiatry.

[20] Hall CL, Walker GM, Valentine AZ, Guo B, Kaylor-Hughes C, James M, Daley D, Sayal K, Hollis C (2014). Protocol investigating the clinical utility of an objective measure of activity and attention (QbTest) on diagnostic and treatment decision-making in children and young people with ADHD—‘assessing QbTest utility in ADHD’(AQUA): a randomised controlled trial. BMJ Open.

[21] Hollis Chris, Hall Charlotte L., Guo Boliang, James Marilyn, Boadu Janet, Groom Madeleine J., Brown Nikki, Kaylor‐Hughes Catherine, Moldavsky Maria, Valentine Althea Z., Walker Gemma M., Daley David, Sayal Kapil, Morriss Richard, Curran Sarah, Clarke Julie, Holsgrove Samina, Jennings Teresa, Kulkarni Neeta, Moldavsky Maria, Nathan Dilip, Skarstam Anne‐Marie, Selby Kim, Vijayan Hena, Williams Adrian (2018). The impact of a computerised test of attention and activity (QbTest) on diagnostic decision‐making in children and young people with suspected attention deficit hyperactivity disorder: single‐blind randomised controlled trial. Journal of Child Psychology and Psychiatry.

[22] Groom MJ, Young Z, Hall CL, Gillott A, Hollis C (2016). The incremental validity of a computerised assessment added to clinical rating scales to differentiate adult ADHD from autism spectrum disorder. Psychiatry Res.

[23] Vogt C, Shameli A (2011). Assessments for attention-deficit hyperactivity disorder: use of objective measurements. Psychiatrist.

[24] Vogt C, Williams T (2011). Early identification of stimulant treatment responders, partial responders and non-responders using objective measures in children and adolescents with hyperkinetic disorder. Child Adolesc Mental Health.

[25] Craig P, Dieppe P, Macintyre S, Michie S, Nazareth I, Petticrew M (2008). Developing and evaluating complex interventions: the new Medical Research Council guidance. BMJ..

[26] Swanson JM, Kraemer HC, Hinshaw SP, Arnold LE, Conners CK, Abikoff HB, Clevenger W, Davies M, Elliott GR, Greenhill LL (2001). Clinical relevance of the primary findings of the MTA: success rates based on severity of ADHD and ODD symptoms at the end of treatment. J Am Acad Child & Adolesc Psychiatry.

[27] Goodman R (1997). The strengths and difficulties questionnaire: a research note. J Child Psychol Psychiatry.

[28] Shaffer D, Gould MS, Brasic J, Ambrosini P, Fisher P, Bird H, Aluwahlia S (1983). A children's global assessment scale (CGAS). Arch Gen Psychiatry.

[29] Guy W. CGI clinical global impressions. EC-DEU Assessment Manual for Psychopharmacology. 1976:76–338.

[30] Wille N, Badia X, Bonsel G, Burström K, Cavrini G, Devlin N, Egmar A-C, Greiner W, Gusi N, Herdman M (2010). Development of the EQ-5D-Y: a child-friendly version of the EQ-5D. Qual Life Res.

[31] Stevens K: The child health utility 9D (CHU9D). A new, paediatric, preference-based measure of health related quality of life PRO newsletter*.* 2010, 43:11–12.

[32] Hill P, Taylor E (2001). An auditable protocol for treating attention deficit/hyperactivity disorder. Arch Dis Childhood.

[33] Beecham J, Knapp M (2001). Costing psychiatric interventions: Gaskell in measuring mental health needs.

[34] Group MC (1999). A 14-month randomized clinical trial of treatment strategies for attention-deficit/hyperactivity disorder. Arch Gen Psychiatry.

[35] Vale C, Fitzgibbon J, Hanley B: Public involvement in clinical trials: supplement to the briefing notes for researchers. http://www i*nvo org uk/wpcontent/uploads/2012/04/INVOLVEpublicinvolvementinclinicaltrialsBriefingnotes2012 pdf]* 2012. Accessed 18 Mar 2018.

[36] Mann C, Chilcott S, Plumb K, Brooks E, Man M-S (2018). Reporting and appraising the context, process and impact of PPI on contributors, researchers and the trial during a randomised controlled trial - the 3D study. Research Involvement and Engagement..

